# The Influence of Self-Serving Leadership on Deviant Behaviors in the Workplace: A Moderated Mediation Model

**DOI:** 10.3389/fpsyg.2022.825154

**Published:** 2022-04-05

**Authors:** Liangcan Liu, Zhitao Wan, Yanping Lin, Xu Wang

**Affiliations:** ^1^School of Business Administration, Guizhou University of Finance and Economics, Guiyang, China; ^2^Personnel Division, Guizhou University, Guiyang, China

**Keywords:** self-serving leadership, organizational identification, moral identity, interpersonal deviance, organizational deviance

## Abstract

Self-serving leadership is a typical example of destructive leadership that has negative effects on its subordinates and organization. According to social identity theory, we propose a theoretical model that self-serving leadership induces employee interpersonal deviance and organizational deviance through organization identification, and we explore the moderating role of moral identity in this relationship. Based on survey data collected from 377 questionnaires by using a three-wave time lagged design, structural equation modeling results showed that (1) there was a significant positive correlation between self-serving leadership and employees’ deviant behavior, (2) organizational identification partially mediates the relationship between self-serving leadership and employees’ deviant behavior, and (3) employees’ moral identity negatively moderates the relationship between self-serving leadership and employees’ organizational identification. The findings further extend the research on the influence of self-serving leadership on employee workplace deviance. They also reveal the mechanisms and boundary conditions of the effect of self-serving leadership on employee workplace deviance.

## Introduction

Since the beginning of organizational behavior research, theory and research have focused on constructive leadership and its effects (Rafferty and Restubog, 2011). Researchers have focused mainly on identifying the characteristics or behaviors of leaders that produce positive results, such as strong work performance, project success, and employees’ innovative behavior. Leadership and its effects have both constructive and destructive elements. Further, negative leadership has a more significant impact on organization members’ behavior compared to positive leadership (Jiang and Gu, 2016). In recent years, as reports on the “dark side” of leadership and leadership behavior have increased, self-serving leadership has begun to attract academic attention. Self-serving leadership is a typical example of destructive leadership (Schmid et al., 2017), in which a leader prioritizes their own needs and interests over the needs of their subordinates and the organization’s goals (Camps et al., 2012). In management practice, leaders do not always think of collective interests (Rafferty and Restubog, 2011), and they often use organizational resources to advance the personal purpose (Camps et al., 2012).

Existing research indicates that self-serving leadership has many adverse consequences for teams and their members (Schyns and Schilling, 2013). Current studies focus mainly on the mechanisms and negative effects of self-serving leadership on employees from the perspectives of social exchange theory and social cognition theory. From the social exchange perspective, self-serving leadership can disrupt the balance of costs and benefits with employees, lead to psychological trauma (Camps et al., 2012), make employees distrust their leaders and destroys cooperation based on trust (Decoster et al., 2021), reduce affective commitment to supervisors (Mao et al., 2019b), cause negative emotions among employees (Camps et al., 2012), induce employees’ desire for retaliation and supervisor-directed deviance (Decoster et al., 2021), and encourage employees’ counterproductive work behavior (Mao et al., 2019b) and deviance from leaders’ instructions (Schyns and Schilling, 2013). Additionally, to restore the exchange balance, employees will inhibit their willingness to cooperate (Decoster et al., 2014), reduce their satisfaction with leaders and their organizational citizenship behavior, increase their turnover intentions (Ritzenhöfer et al., 2019), decrease their motivation to voice (Liu et al., 2017). At the team level, studies have revealed that self-serving leadership harms team performance (Mao et al., 2019a) and destroys team creativity and knowledge sharing (Peng et al., 2019). From the social cognitive perspective, self-serving leadership will arouse employees’ uncertainty about their outcomes, leading them to experience negative emotions (Camps et al., 2012). Moreover, when employees ascribe selfishness to a leader, it will reduce their satisfaction and OCB toward the leader and increase their intentions to abandon the leader (Ritzenhöfer et al., 2019). Meanwhile, viewed within the cognitive-affective processing system framework (Mischel and Shoda, 1995), self-serving leadership triggers moral disengagement and negative emotions among employees, thus producing deviant behavior (Zhou et al., 2021). Concerning the boundary conditions of self-serving leadership’s negative impact on employees, existing research indicates that organizational budget policy (Decoster et al., 2014), ethical climate (Decoster et al., 2021), employee perceptions of distributive justice (Camps et al., 2012), employee power distance orientation (Mao et al., 2019b), and justice sensitivity (Zhou et al., 2021) are important contingent factors that influence the relationship between self-serving leadership and employee behavior. As an essential reference point for employee behavior, a supervisor’s behavior is important in shaping employee behavior (Sulea et al., 2013). Deviance at work is a harmful extra-role behavior that is intentionally carried out by employees, violates organizational principles, and poses a menace to the organization and its members (Bennett and Robinson, 2000). It is typical negative behavior in the workplace. It can bring substantial economic losses to enterprises (Robinson and Bennett, 1995).

Existing research has shown that there are complex cognitive and emotional mechanisms (e.g., moral disengagement, anger, etc.), underlying self-serving leadership’s effect on employees’ deviant behaviors (Zhou et al., 2021). However, beyond these mechanisms, we must also explore other cognitive factors (e.g., organizational identification) and emotional factors (e.g., workplace anxiety). Moreover, existing research explains the mechanism of self-serving leadership mainly using social exchange theory and social cognition theory, and it is necessary to consider other theoretical orientations (e.g., social identity theory). Therefore, this study introduces organizational identification as a mediating variable to better understand why self-serving leadership leads to employees’ deviant behavior and appeals to social identity theory to explain this mediating mechanism. Meanwhile, individual characteristics determine the degree or direction of leadership influence (Zhang and Liu, 2018). Specifically, employees with different characteristics may have different reactions to the same leadership behavior. Additionally, culture plays an important role in shaping individuals’ beliefs, opinions, attitudes, and behaviors (Hofstede, 2001). Therefore, this paper also examines the contingent effect of differences in employees’ moral identity on the relationship between self-serving leadership and employee deviant behavior in the Asian cultural context.

Ashworth and Mael introduced social identity theory into the field of organizational behavior research. Social identity theory (Turner et al., 1987) holds that when an organization meets employees’ needs for security, self-realization, and belonging, individuals classify themselves as members of that organization, which enhances employees’ identification with and emotional attachment to the organization (Blader et al., 2017) and encourages employees to defend the organizational interests (Van Dick et al., 2006). However, when employees’ organizational identification is weak, they distance themselves from the organization and are indifferent to its interests, which can easily produce behaviors that are not beneficial to the organization.

Self-serving leadership conveys harmful intentions to subordinates, making subordinates afraid of being exploited and hesitant to take risks (Liu et al., 2017), as well as fearful of uncertainty (Camps et al., 2012). Because the supervisor is the spokesperson of the organization, their behavior also represents the organization’s attitude towards employees. Therefore, employees who suffer from self-serving leadership will lowering their emotional commitment (Mao et al., 2019a) and have weaker organizational identification (Liu et al., 2017), encouraging workplace deviance.

Therefore, In light of social identity theory, this research will explore an intermediate process in which self-serving leadership influence employee deviance via organizational identification. Furthermore, in order to reveal the relationship more comprehensively, this study will further explore the moderated effect of moral identity in this relationship. Based on the self-regulation mechanism of moral behavior, moral identity take important effect on understanding employees’ moral behavior (Watts and Ronald Buckley, 2017). Moral identity is “a self-concept formed based on a series of moral characteristics,” which describes individual differences in moral terms (Aquino and Reed, 2002). It is an important boundary condition affecting individuals’ deviant behavior (Mulder and Aquino, 2013). We hypothesize that the negative effects of self-serving leadership on employees’ organizational identification are also impacted by their level of moral identity. Individuals with high moral identification have more likelihood of noticing morality-related information (DeCelles et al., 2012). When employees perceive that their moral values are consistent with the ethical norms of the organization, they will enhance their identification with the organization (Wang et al., 2017). According to this logic, when employees and organization have different ethical norms, it will have a negative impact on employees’ identification with the organization. We consider the effects may be complicated. Different cultures affect subordinates’ views of and reactions to leaders’ behaviors (Zhang and Liu, 2018). The strength of individuals’ moral identity is not only affected by their level of moral cognition but also restricted by situational factors (Shao et al., 2011). Due to the legitimacy of managers’ hostility to subordinates, maintaining hierarchical status and respect for authority is characteristics of Asian culture (Zhang and Liu, 2018). We surmise that higher moral identities of employees may buffer the detrimental influence of self-serving leadership on their organizational identification in an Asian cultural context.

**FIGURE 1:**
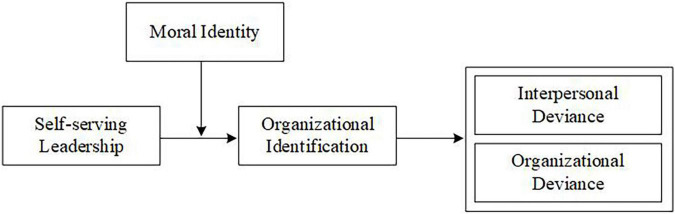
Theoretical model.

In terms of social identity theory, this study concentrate on how self-serving leadership exert influence on employees’ deviance in the workplace, explores the mediating effect of organizational identification, and analyzes how moral identity modifies it, as well as makes two major theoretical contributions. First, this research reveals the influencing mechanism of self-serving leadership on employees’ workplace deviant behavior in terms of organizational identification and enriches leadership theory. Second, this study introduces moral identity in Asian culture to illustrate the link between self-serving leadership and organizational identification, making some contributions to the localization research of self-serving leadership (see Figure 1).

## Literature Review and Hypotheses

### Self-Serving Leadership and Deviant Behavior in the Workplace

Self-serving leaders will prioritize their own interests over those of their subordinates and organizations (Camps et al., 2012), which have negative impact on the organization and subordinates (Haynes et al., 2015). Following this logic, we hypothesize that as a typical negative behavior in the workplace (Kluemper et al., 2013), employee deviance may be induced by self-serving leadership, including interpersonal deviance toward the leader as the source of aggression as well as toward innocent colleagues, and organizational deviance such as retaliation against the organization (Kluemper et al., 2015). First, leadership style can positively impact organizational ethical climate (Grojean et al., 2004), which affects employees’ behaviors (Wimbush and Shepard, 1994). According to social learning theory (Bandura, 1977), individuals learn by observing and imitating role models’ behaviors. Employees who are exposed to self-serving leaders observe self-serving behaviors, acquire self-serving values, and then engage in self-serving behaviors themselves (Haynes et al., 2015), and avoid prosocial behavior (Liu et al., 2017). Therefore, self-serving leadership creates an organizational climate in which members’ putting their own interests first is acceptable and will not be punished (Peng et al., 2019), leading employees to follow self-interested cognitive and behavioral norms (Vardaman et al., 2014) and engage in unethical behavior (Wimbush and Shepard, 1994). Second, according to negative reciprocal norms, there is a need to eliminate the imbalance of exchange between the two sides, achieve self-protection (Biron, 2010), and address perceptions of injustice (Mitchell and Ambrose, 2007). The party who is treated unfairly may behave negatively toward self-serving leaders (Camps et al., 2012). When there is a conflict of interests between leaders and subordinates, self-serving leaders may pursue their interests at the expense of subordinates (Peng et al., 2019) and even attribute their subordinates’ achievements to themselves (Schmid et al., 2017). In confront of this situation, subordinates feel a sense of threat that their resources are expended (Mao et al., 2019a) and that there is a gap between their efforts and expected benefits. In turn, this can make subordinates inclined to take revenge to restore the balance (Carlsmith et al., 2002). Some studies have also shown that employees seek revenge against the person who has wronged them to reestablish a feeling of fairness and avoid future aggression (Aquino and Thau, 2008; De Dreu and Nauta, 2009). Simultaneously, when leaders lack sympathy for or even exploit their subordinates, subordinates perceive that their interests are threatened (Peng et al., 2019), their psychological safety is harmed, and they experience negative emotions, such as intense fear (Peng et al., 2019). Subordinates usually take specific actions (Tice and Bratslavsky, 2000) to address negative emotions, with deviant behavior toward their leader (Spector and Fox, 2002). In addition, the more employees trust their organization and its leaders, the more they will feel attached to the organization and its members and the less they will engage in deviant behaviors (Bennett and Stamper, 2001). Self-serving leaders violate employees’ expectations, cause crises of trust, and engender employees’ desire for revenge (Decoster et al., 2021). Therefore, in response to leaders’ self-serving behavior and the need to prevent further threats, employees who believe they are being treated unequally will intentionally deviate from leaders’ instructions to retaliate. Furthermore, in addition to deviant behavior directed at supervisors, employees may also engage in deviant behavior toward their colleagues. According to the theory of displaced aggression (Burton et al., 2012), due to certain inhibitory factors, frustrated individuals cannot directly vent their emotions to the source of frustration and instead must attack surrogate objects. Due to the power imbalance between leaders and employees, deviant behaviors toward leaders may cause employees to suffer punishment and counter-retaliation, such as losing opportunities for salary increases or promotions. Therefore, there is a risk of deviant behavior directed at leaders. To avoid the possible danger, employees may direct their hostility or deviance toward targets with less obvious status differences, such as colleagues.

Simultaneously, due to the dual representation of leaders, leaders are often regarded as agents of organizations. Leader-member exchange entails social exchange both between leaders and employees and in the “spillover effect” between organizations and employees (Rupp and Cropanzano, 2002). If self-serving leaders pursue their own interests at the expense of their subordinates, employees may interpret this as the organization’s encroachment on their interests, regard the leaders’ self-serving behaviors as behaviors representing the organization, and then follow negative reciprocal norms and engage in deviant behaviors. Additionally, the organization has moral and legal responsibility for leaders’ behavior (Shoss et al., 2013). Therefore, employees will attribute leaders’ self-serving behavior to the organization to some extent, which will have a detrimental impact on organization-member exchange, as a consequence, employees are more inclined to participate in deviant behaviors that are destructive to the organization. We thus propose the following hypotheses.

H1a: Self-serving leadership is positively correlated with interpersonal deviance.

H1b: Self-serving leadership is positively correlated with organizational deviance.

### Mediating Role of Organizational Identification

Social identity theory (Turner et al., 1987) holds that when the organization meets employees’ needs for security, self-realization, and belonging, individuals will classify themselves as members of that organization. Such classification will improve employees’ identity, foster attachment to the organization (Blader et al., 2017), and make employees act to support the organization (Van Dick et al., 2006). As a special form of social identification, organizational identification is defined as individuals taking themselves as part of the organization and thus belonging to it. As an important part of organizational context, leaders’ behavior significantly impacts employees’ identity (He and Brown, 2013). Self-serving leadership can be considered an unethical leadership behavior (Peng et al., 2019), in which actions are beneficial to leaders themselves and taking advantage of others’ interests (DeCelles et al., 2012). Typical examples include leaders diverting scarce resources toward themselves, pilfering recognition from subordinates, and evading responsibility (Rus et al., 2010). In the organizational context, self-serving leadership is more common than hostile and aggressive behaviors associated with abusive supervision (Schmid et al., 2017). Studies show that subordinates will interpret leaders’ self-serving behaviors as hostile, perceiving the risk of exploitation (Liu et al., 2017), reducing their emotional commitment, and producing more counterproductive behaviors (Mao et al., 2019b). Accordingly, we hypothesize that self-serving leadership will reduce employees’ identification with the organization.

First, an important mission of leaders is to motivate subordinates to contribute to the organization by influencing their self-concept (van Knippenberg et al., 2004), which is a significant component of identity (Tajfel and Turner, 1986). An effective leader should transform the subordinate’s identity from self-oriented to group-oriented, but a leader’s selfish behavior will hinder this process (Epitropaki et al., 2017). Self-serving leadership also weakens employees’ trust in leaders (Decoster et al., 2021). As supervisors are the organization’s spokespeople, their behavior also represents organizations’ attitudes towards employees (Aryee et al., 2007). When leaders are self-serving, this will make employees are less emotionally attached to their organization (Mao et al., 2019a). Second, self-serving leadership threatens subordinates’ perception of control. Individuals tend to maintain control over the surrounding environment to reduce uncertainty (Friedland et al., 1992). In the organizational environment, the leader is responsible for allocating resources, and their selfish behavior damages subordinates’ resources (Mao et al., 2019a). When faced with selfish leaders, subordinates will feel more uncertainty, less control over personal achievements (Camps et al., 2012), and more helplessness (Rothbaum et al., 1982), leading to the decline of organizational identification. Finally, as the main object of employees’ interpersonal communication in the organization (Tekleab and Taylor, 2003), employees expect a safe work environment, respect, and fairness (Salin and Notelaers, 2017). Leaders’ self-serving behavior makes subordinates feel that their interests (i.e., respect and fairness) are violated, leading employees to feel excluded by the organization and harming their sense of identity to the organization.

Organizational identification is an important predictor of employees’ attitudes and behavior (Tajfel and Turner, 1986). Strong organizational identification can inhibit deviant behavior. When the higher the organizational identification employees are, the stronger awareness of being part of the organization for theirs (Ashforth and Mael, 1989) and strongly associate organizational goals with personal development, stimulating their initiative (Dutton et al., 1994) and promoting group achievements. This is true even if their contributions and efforts are not encouraged by the organization’s salary system (Organ, 1988). Employees with strong organizational identification have strong collective awareness (Brewer and Gardner, 1996). When collective interests conflict with individual interests, these employees are willing to prioritize the organization, effectively reducing deviance. These employees also integrate personal identity and organizational identification, which can strengthen cooperation to achieve organizational goals and reduce interpersonal conflict (Liu and Phillips, 2011). Therefore, employees with strong organizational identification actively contribute to their organization and avoid interpersonal conflicts that are unfavorable to the organization, mitigating interpersonal deviance.

According to the analysis above, we surmise that self-serving leadership’s effect on employees’ deviance may occur through the mediator of organizational identification. When people recognize they’re a part of a particular group, they will also realize the emotional significance and value brought to them by other members of the group (Sillince and Golant, 2018), encouraging employees to support the organization. Self-serving leadership makes employees recognize the gap between their efforts and expected returns, leads to perceptions of being exploited, reduces emotional attachment to the organization, and then stimulates employees to exhibit interpersonal and organizational deviant behaviors in social exchanges. Consequently, we propose the following hypotheses.

H2a: Employee organizational identification mediates the effect of self-serving leadership on interpersonal deviance.

H2b: Employee organizational identification mediates the effect of self-serving leadership on organizational deviance.

### Moderating Effect of Moral Identity

As an important composition factor of self-concept, moral identity is formed by some typical moral qualities of an individual (e.g., kindness, compassion, fairness, friendliness, generosity, diligence, helpfulness, honesty, etc.; Aquino and Reed, 2002). It consists of two aspects. First, internalization refers to the stability of moral characteristics in the self-concept or self-schema. Second, symbolization refers to the degree of expression of moral characteristics in moral behavior (Aquino and Reed, 2002). An individual’s moral identity has a positive impact on inducing their internal motivation (He et al., 2014), which has a self-regulating mechanism and can regulate an individual’s attitudes and behavior (Skubinn and Herzog, 2016). Employees’ moral identity may mitigate the negative effects of self-serving leadership on employees’ organizational identification. On the one hand, research has shown that employees with strong moral identity can build positive interpersonal relationships. Individuals with strong moral identity are more inclusive (Choi and Winterich, 2013), ready to reorient their focus from themselves to others (Moshman, 2011), which establishes trust between employees and leaders (Dutton et al., 2010) and promotes a high level of leader-member exchange (Graen and Uhl-Bien, 1995). This is important for several reasons. First, high-quality relationships between leaders and followers increase employees’ tolerance for injustice, allow them to focus on more positive factors, and urge them to return goodwill in different ways (Kamdar and Van Dyne, 2007). Second, high-quality relationships improve reciprocal exchange among group members (Hantula, 2009) and promote open communication between leaders and employees (Vinarski-Peretz et al., 2011), enhance mutual understanding (Liao et al., 2010), give employees a more positive outlook on the behavior of others in the organization (including self-serving leadership), and make employees believe that leaders’ benefit distribution is reasonable. On the other hand, different cultures emphasize different norms of interpersonal communication, and subordinates’ environment affects their views of and reactions to leaders’ behaviors (Zhang and Liu, 2018). The strength of individuals’ moral identity is not only affected by their level of moral cognition but also restricted by situational factors (Shao et al., 2011). Culture can influence individuals’ level of moral cognition (Mikula and Wenzel, 2000), thus affecting individuals’ moral identity (Shao et al., 2011), leading to differences in moral judgments and evaluations of the same phenomenon (Zhang and Liu, 2018). In Western cultures, employees are expected to be treated with dignity and respect by their supervisors (Hofstede, 2001). Individuals with strong moral identity pay more attention to morally relevant information (DeCelles et al., 2012), and have stronger awareness of cognitive processing of such information (Eisenbeiss and Knippenberg, 2015), and are more aware of moral problems and responsive to behaviors that violate social norms (Aquino and Reed, 2002). The more strongly subordinates perceive their leaders’ behaviors as violating moral norms, the more strongly they perceive injustice from their superiors (Zhang and Liu, 2018), losing trust in leaders (Decoster et al., 2021), experiencing negative emotions (Camps et al., 2012), and sometimes engaging in retaliatory behavior (Decoster et al., 2021). In contrast, Asian cultures uphold the legitimacy of managers’ hostility to subordinates, promoting hierarchical status and respect for authority (Zhang and Liu, 2018). Some scholars studied the relationship between ethical leadership and moral disengagement based on samples from China and the United States, and found that moral identity has different moderating effects. They consider that the culture may explain the difference (Moore et al., 2019). Therefore, we argue that in Asian cultures, subordinates may not regard leaders’ self-serving behaviors as immoral, which makes employees with strong moral identity less sensitive to moral cues in organizational situations and mitigates employees’ negative emotions (including feelings of injustice).

Accordingly, we can speculate that moral identity can take a moderating role between self-serving leadership and organizational identification. Employees with high moral identity prefer to establish trust with leaders and colleagues, have high-quality leader-member exchange, improve the collection and processing of leadership information, and pay greater attention to the favorable aspects of leadership, reducing the disadvantageous influence of self-serving leadership on organizational identification. Meanwhile, in Asian cultures, subordinates with strong moral identity are less sensitive to moral cues of leadership, even to their leaders’ unethical behavior. On the contrary, employees with weak moral identity cannot establish trusting relationships with their leaders and colleagues, maintaining a low level of leader-member exchange. They can only rely on simple clues to form an understanding of leaders’ behavior. Therefore, the negative impact of self-serving leadership on employees’ organizational identification is amplified by a more unfavorable impression of a leader’s behavior. We propose the following hypotheses.

H3: Moral identity mitigates the negative effect of self-serving leadership on employees’ organization identification; for employees with low moral identity, this effect is stronger than for those with high moral identity.

Moreover, we present a moderated mediation model of self-serving leadership affecting employees’ deviant behavior. As shown in Figure 1, under the influence of self-serving leadership’s self-identity orientation to employees and the perception that their own interests have been violated, employees with weak moral identity are more sensitive to the moral information contained in self-serving leadership. Leadership represents the organization (Aryee et al., 2007); therefore, for employees with weak moral identity, self-serving leadership has a greater negative impact on employees’ organizational identification and is more likely to lead to deviant behavior. However, in Asian cultural contexts, employees with strong moral identity will be less sensitive to leaders’ moral cues. Meanwhile, employees with strong moral identity easily establish high-quality leader-member exchange, such that employees pay more attention to leaders’ positive qualities. Therefore, employees with strong moral identity have a more positive evaluation of self-serving leadership, which alleviates its negative impact on employees’ organizational identification and makes employees less likely to engage in deviant workplace behavior. As a consequence, we posit the final hypotheses.

H4a: Moral identity moderates the indirect effect of self-serving leadership on interpersonal deviance through organizational identification, such that the indirect effect is weaker when moral identity is higher.

H4b: Moral identity moderates the indirect effect of self-serving leadership on organizational deviance through organizational identification, such that the indirect effect is weaker when moral identity is higher.

## Materials and Methods

### Sample and Procedure

Data were collected from full-time employees of various organizations located in china, involving finance, education, healthcare, information technology, manufacturing, retail and other industries. We administered surveys via mail. We asked Master of Tourism Administration (MTA) and Master of Business Administration (MBA) students from a university in western China to serve as organizational contacts in exchange for course credit. All the students were working full-time. We instructed them to invite three to four subordinates under their direct management or three to four colleagues who were willing to participate. This convenience sampling technique has been used successfully by a variety of researchers (Greenbaum et al., 2012; Mitchell et al., 2015). Ultimately, we received contact information for 441 potential study participants.

The study featured three phases of data collection to limit common method bias (Podsakoff et al., 2012). We deemed 2 weeks to be an appropriate interval between phases (for a similar approach, see Eva et al., 2020). Additionally, to match participants’ questionnaires for the three phases, we assigned codes for the participants invited by the students and marked them on the envelopes containing the notes and questionnaires before administration.

To improve participants’ motivation and increase the questionnaire response rate, we took three measures. First, the researchers emphasized to students and participants that the survey data were for research purposes only and would be completely confidential. To ensure confidentiality, we placed a strip on each envelope that was sealed after the questionnaire was complete. Second, we provided participants with an inexpensive but practical gift. Third, the research activities had a practical incentive for student in that they would receive course credit only upon their acquaintance’s completion of the questionnaire.

At Time 1 (T1), We ask participants to evaluate their perceived leadership style and moral identity and to provide personal background information. We issued 441 questionnaires, of which 436 were returned (recovery rate = 98.9%). At Time 2 (T2), approximately 2 weeks later, participants evaluated organizational identification. We issued 436 questionnaires at T2, of which 433 were returned (recovery rate = 99.3%). At Time 3 (T3), approximately 2 weeks after that, participants evaluated workplace deviant behavior. We issued 433 questionnaires at T3, of which 431 were returned (recovery rate = 99.5%). After all data collection, we screened the questionnaires, eliminating ones with regular answers and many missing data for the main variables. Finally, we obtained 377 valid questionnaires, an effective rate of 87.07%. The descriptive characteristics of the samples are shown in Table 1.

**TABLE 1:** Descriptive characteristics of samples (*N* = 377).

Characteristic	Category	Number	Percentage (%)	Characteristic	Number	Number	Percentage (%)
Gender	Male	150	39.8	Age	≤25 years	39	10.3
	Female	227	60.2		26 ∼35 years	262	69.5
Education	Junior high school degree or below	1	0.3		36 ∼45 years	58	15.4
	High school or vocational high school degree	14	3.7		>45 years	18	4.8
	Junior college and Bachelor degree	300	79.6				
	Master degree	62	16.4	Time working with supervisor	<2 years	180	47.7
Tenure	≤5	211	56		2 ∼5 years	124	32.9
	6∼10	116	30.8		6 ∼10 years	60	15.9
	11 ∼20	33	8.8		>10 years	13	3.4
	≥21	17	4.5				

### Measures

Since the measurements adopted in our research were created in Western countries, we employed a translation and back-translation method (Brislin, 1970) to ensure the reliability and validity of their Chinese versions. The final survey was formed through several rounds of group discussions. Except for demographic variables, all measures used Likert five-point scoring (1 = “strongly disagree” and 5 = “strongly agree”).

### Self-Serving Leadership (SL)

We assessed SL using a four-item scale borrowed by Camps et al. (2012). Participants rated their agreement with statements about their direct supervisor’s behavior (e.g., “My superior does not show consideration for his/her followers, only for him/herself”). Cronbach’s α for this scale was 0.93.

### Moral Identity (MI)

We assessed MI using a five-item scale that was suggested by Aquino and Reed (2002). Participants were shown nine moral characteristics that they may use to characterize themselves (e.g., caring, compassionate, fair, generous) and assess how much they agreed with a set of statements concerning their internalization of these characteristics (e.g., “It would make me feel good to be a person who has these characteristics”). Cronbach’s α for this scale was 0.89.

### Organizational Identification (OI)

We assessed OI base on the six-item scale adopted by Mael and Ashforth (1992). Participants rated their agreement with statements about their identification with the organization (e.g., “When I talk about my organization, I usually say ‘we’ rather than ‘they”’). Cronbach’s α for this scale was 0.88.

### Workplace Deviant Behavior (WDB)

We assessed WDB using a 12-item scale developed by Liao et al. (2004). Six items assessed perceptions of organizational deviance (WDB-O). Respondents indicated behaviors targeted at their current company (e.g., “damaged property belonging to your employer”). The remaining six items assessed perceptions of interpersonal deviance (WDB-I). Respondents indicated behaviors targeted at coworkers (e.g., “publicly embarrassed someone at work”). Cronbach’s α values for interpersonal deviance and organizational deviance were 0.91 and 0.86, respectively.

### Control Variables

Following other studies (Aquino and Douglas, 2003; Berry et al., 2007), we assessed employees’ age, gender, education, tenure, and time working with their current direct supervisor as control variables.

## Results

### Confirmatory Factor Analysis

We constructed six models and conducted confirmatory factor analyses (CFAs) adopting Amos 24 to evaluate discriminate validity for the hypothesized model. The results of CFA given in Table 2 indicate that the proposed five-factor model fitted the indices well (*χ^2^* = 770.76, df = 314, CFI = 0.93, TLI = 0.92, RMSEA = 0.06, SRMR = 0.04). This showed that the five core constructs of this study (self-serving leadership, organizational identification; moral identity; interpersonal deviance; organizational deviance) all had a good discrimination validity. Additionally, when items’ standardized factor loadings exceed 0.6 (Bagozzi, 1981), composite reliability (CR) exceeds 0.7, and average variance extracted (AVE) exceeds 0.5, the scale is considered to have good convergent validity (Fornell and Larcker, 1981). As shown in Table 3, the standardized factor loadings of most survey items exceeded 0.6 (MI-3 = 0.56), and the CR and AVE values of each dimension met the criteria, indicating that the scale had good convergent validity. Meanwhile, we adopt the heterotrait–monotrait ratio (HTMT) created by Henseler et al. (2015) to test the discriminant validity.

**TABLE 2:** Confirmatory factor analysis and model comparison.

Model	*χ^2^*	df	Δχ^2^/Δ df	CFI	TLI	RMSEA	SRMR
1. Five factors: SL; OI; ML; WDB-I; WDB-O	770.76	314	–	0.93	0.92	0.06	0.04
2. Four factors a: SL; OI; ML; WDB-I+WDB-O	982.93	318	212.17 (4)***	0.90	0.89	0.08	0.05
3. Four factors b: SL; OI+ML; WDB-I; WDB-O	1793.31	318	1022.55 (4)***	0.78	0.76	0.11	0.13
4. Three factors: SL+OI+ML; WDB-I; WDB-O	2835.45	321	2064.69 (7)***	0.62	0.59	0.14	0.16
5. Two factors: SL+OI+ML; WDB-I+WDB-O	3046.30	323	2275.54 (9)***	0.59	0.56	0.15	0.16
6. Single factors: SL+OI+ML+WDB-I+WDB-O	4014.04	324	3243.28 (10)***	0.45	0.40	0.17	0.16

N = 377. SL, self-serving leadership; OI, organizational identification; MI, moral identity; WDB-I, interpersonal deviance; WDB-O, organizational deviance, ***p < 0.001.

**TABLE 3:** Convergent validity and discrimination validity analysis.

Variable	Items	Item reliability	Composite reliability	Convergence validity
		STD.LOADING	CR	AVE
1. SL	4	0.80—0.94	0.93	0.76
2. OI	6	0.66—0.81	0.89	0.56
3. MI	5	0.56—0.89	0.89	0.62
4. WDB-I	6	0.73—0.88	0.92	0.65
5. WDB-O	6	0.69—0.78	0.88	0.54

*N = 377. SL, self-serving leadership; OI, organizational identification; MI, moral identity; WDB-I, interpersonal deviance; WDB-O, organizational deviance.*

Compare with traditional discriminant validity assessment methods, heterotrait–monotrait ratio (HTMT) approaches are more reliable to detect discriminant validity issues (Henseler et al., 2015). The calculation HTMT ratio was conducted by using the plugin specially developed for AMOS 24 by Gaskin et al. (2019). If the value of the HTMT is higher than the threshold of 0.85, we conclude that there is a lack of discriminant validity (Henseler et al., 2015). As shown in Table 4, the HTMT value between each pair of factors lower than 0.85, indicates the five variables involved in this study are distinguishable. Further, the study adopted three phases to collect data, effectively controlling for common method bias (Siemsen et al., 2009).

**TABLE 4:** Analysis of HTMT discriminant validity.

	MI	OI	SL	WDB-I	WDB-O
MI					
OI	0.24				
SL	0.14	0.28			
WDB-I	0.23	0.26	0.35		
WDB-O	0.20	0.32	0.39	0.83	

*Heterotrait-Monotrait Ratio (HTMT); SL, self-serving leadership; OI, organizational identification; MI, moral identity; WDB-I, interpersonal deviance; WDB-O, organizational deviance.*

### Descriptive Statistics and Correlations

Table 5 shows means, standard deviations, inter-correlations, and alpha coefficient of main research variables. The diagonal shows the measures’ internal consistency coefficients.

**TABLE 5:** Means, standard deviations, and correlations.

Variable	M	SD	1	2	3	4	5	6	7	8	9	10
1. Sex T1	0.40	0.49										
2. Age T1	2.15	0.65	0.13**									
3. Education T1	3.12	0.44	0.03	−0.16**								
4. Tenure T1	1.62	0.83	0.07	0.62**	−0.16**							
5. Time working with supervision T1	1.75	0.85	0.07	0.39	−0.15**	0.53**						
6. SL T1	1.76	0.97	0.05	0.05	0.15**	–0.01	0.02	(0.93)				
7. MI T1	4.34	0.71	–0.07	–0.04	0.02	–0.04	0.03	−0.13*	(0.89)			
8. OI T2	3.87	0.75	0.01	–0.06	–0.04	–0.06	–0.09	−0.25**	0.21**	(0.88)		
9. WDB-I T3	1.33	0.58	0.05	0.02	0.02	0.07	0.02	0.32**	−0.21**	−0.23**	(0.91)	
10. WDB-O T3	1.24	0.46	0.08	0.01	0.06	0.04	0.02	0.34**	−0.17**	−0.28**	0.74**	(0.86)

N = 377; Reliability estimates are reported in the diagonal. SL, self-serving leadership; OI, organizational identification; MI, moral identity; WDB-I, interpersonal deviance; WDB-O, organizational deviance, *p < 0.05, **p < 0.01. T1 = Time 1; T2 = Time 2; T3 = Time 3.

Self-serving leadership was positively impact on interpersonal deviance (*r* = 0.32, *p* < 0.01) and organizational deviance (*r* = 0.34, *p* < 0.01) and negatively related to organizational identification (*r* = −0.25, *p* < 0.01). Organizational identification was negatively correlated with interpersonal deviance (*r* = −0.23, *p* < 0.01) and organizational deviance (*r* = −0.28, *p* < 0.01).

### Hypothesis Testing

Table 6 summarizes the results of the regression analyses. In Model 4, self-serving leadership was positively impact on interpersonal deviance (β = 0.32, *p* < 0.001), and in Model 7, self-serving leadership was positively impact on organizational deviance (β = 0.34, *p* < 0.001). These results support Hypotheses 1a and 1b.

**TABLE 6:** Hierarchical regression results for the direct and mediation models.

Variable	OI		WDB-I			WDB-O		
	M1	M2	M3	M4	M5	M6	M7	M8
Sex	0.03	0.04	0.05	0.04	0.04	0.08	0.06	0.07
Age	–0.04	–0.01	–0.03	–0.06	–0.06	–0.02	–0.05	–0.06
Education	–0.06	–0.02	0.02	–0.03	–0.03	0.06	0.01	0.01
Tenure	0.00	–0.01	0.10	0.11	0.11	0.06	0.07	0.07
Time working with supervisor	–0.09	–0.08	–0.02	–0.03	–0.05	0.01	–0.00	–0.02
SL		−0.25***		0.32***	0.28***		0.34***	0.29***
OI					−0.17**			−0.21***
F	1.00	4.75**	0.66	7.59***	8.27***	0.90	8.86***	10.57***
R^2^	0.01	0.07	0.01	0.11	0.14	0.01	0.13	0.17
ΔR^2^		0.06		0.10	0.03		0.11	0.04

N = 377. SL, self-serving leadership; OI, organizational identification; MI, moral identity; WDB-I, interpersonal deviance; WDB-O, organizational deviance, **p < 0.01, ***p < 0.001.

We adopted the mediating effect test proposed by Baron and Kenny (1987). First, in Models 4 and 7, self-serving leadership predicted interpersonal deviance and organizational deviance. This satisfied the first criterion for the test. Second, in Model 2, self-serving leadership predicted organizational identification (β = −0.25, *p* < 0.001), satisfying the second criterion. Third, in Models 5 and 8, self-serving leadership and organizational identification predicted interpersonal deviance (β = 0.28, *p* < 0.001 and β = −0.17, *p* < 0.01, respectively) and organizational deviance (β = 0.29, *p* < 0.001 and β = −0.21, *p* < 0.001), satisfying the third criterion. These findings suggested that self-serving leadership indirectly affected employees’ interpersonal and organizational deviance, which occurred through organizational identification. Specifically, self-serving leadership weakens employees’ organizational identification, which induces interpersonal deviance and organizational deviance. This meant that Hypotheses 2a and 2b were supported.

To more rigorously test the mediating effect, we also adopted the bootstrap method recommended by Preacher and Hayes (2008) and utilized the PRODCLIN program of MacKinnon et al. (2007). We analyzed confidence intervals based on 5,000 bootstrap samples (Preacher and Hayes, 2008).

As shown in Table 7, the mediating effects of organizational identification in the relationships of self-serving leadership on interpersonal deviance and self-serving leadership on organizational deviance were 0.02 and 0.02, respectively. The 95% confidence intervals – which were [0.01, 0.04] and [0.01, 0.04], respectively – did not include zero. Using the PRODCLIN program (MacKinnon et al., 2007), we found significant indirect effects of self-serving leadership on interpersonal deviance and organizational deviance via organizational identification (β = 0.1, 95% CI [0.01, 0.04] and β = 0.1, 95% CI [0.01, 0.04], respectively). These results indicated that organizational identification had a significant mediating effect in these relationships, lending further support to H2a and H2b.

**TABLE 7:** The indirect effects of self-serving leadership on dependent variables.

Item	Path	Point estimate/Index		Bootstrapping (95% CIs)		Program PRODCLIN (95% CIs)	
			SE	Lower	Upper	Lower	Upper
Totle effect	SL–WDB-I	0.19	0.03	0.13	0.25		
Direct effect	SL–WDB-I	0.17	0.03	0.11	0.23		
Indirect effect	SL–WDB-I	0.02	0.01	0.01	0.04		
Indirect effect	SL–OI– WDB-I	0.01				0.01	0.04
Totle effect	SL –WDB-O	0.16	0.02	0.12	0.21		
Direct effect	SL–WDB-O	0.14	0.02	0.09	0.18		
Indirect effect	SL–WDB-O	0.02	0.01	0.01	0.04		
Indirect effect	SL–OI—WDB-O	0.01				0.01	0.04

*N = 377. SL, self-serving leadership; OI, organizational identification; MI, moral identity; WDB-I, interpersonal deviance; WDB-O, organizational deviance.*

We multiplied self-serving leadership (centered) and moral identity as an interaction term to verify H3. As shown in Table 8, the regression result from Model 3 indicates that this interaction term significantly predicted organizational identification (β = 0.14, *p* < 0.01).

**TABLE 8:** Hierarchical regression results for the moderation model.

Variable	OI		
	M1	M2	M3
Sex	0.03	0.05	0.05
Age	–0.04	–0.01	–0.01
Education	–0.06	–0.03	–0.01
Tenure	0.00	0.00	0.00
Time working with supervisor	–0.09	–0.10	–0.10
SL		−0.22***	−0.23***
MI		0.19***	0.18***
SL × MI			0.13**
F	1.00	6.31***	6.52***
R^2^	0.01	0.11	0.12
ΔR^2^		0.10	0.02

N = 377. SL, self-serving leadership; OI, organizational identification; MI, moral identity; WDB-I, interpersonal deviance; WDB-O, organizational deviance, **p < 0.01, ***p < 0.001.

**FIGURE 2:**
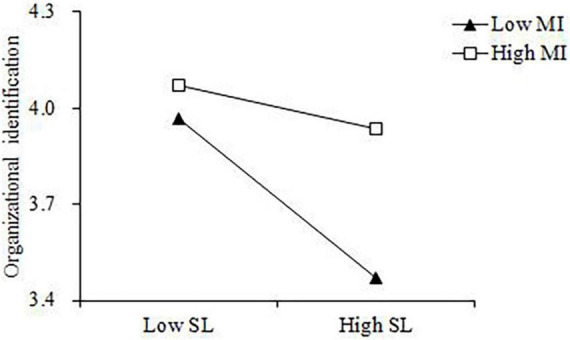
Moderating effect of moral identity between self-serving leadership and employees’ organizational identification.

Moreover, this study adopted simple slope analysis to describe the difference of the impact of self-serving leadership on organizational identification with different levels of moral identity. As plotted in Figure 2, When the employee has a high moral identity, the adverse influence of self-serving leadership on the employee’s organizational identification was weaker. On the contrary, when the employees’ moral identity was weak, self-serving leadership had a strong adverse impact on organizational identification. Thus, further supporting H3.

We further examined the moderated mediation model posited in H4a and H4b by employing the MODMED macro v3.5 (model 7) (Hayes, 2013). First, the dependent variable was regressed with the control variables, independent variable, mediator, moderator, and interaction term. The statistically significant effect of self-serving leadership × moral identity in the regression equations for organizational identification (β = 0.14, *p* < 0.01) implied that moral identity significantly moderated the indirect impact of self-serving leadership on organizational identification. Second, We assess two sets of effects at the high and low levels of the moral identity. The result is presents in Table 9, When employees’ moral identity is low, indirect effects were significant (interpersonal deviance: indirect effect = 0.04, CI [0.02, 0.06], excluding zero; organizational deviance: indirect effect = 0.04, CI [0.02, 0.06], excluding zero) but not significant in the high moral identity condition (interpersonal deviance: indirect effect = 0.01, CI [−0.01, 0.03]; organizational deviance: indirect effect = 0.01, CI [−0.00, 0.03], including zero). The results implying that when employees with low moral identity, the mediating impact of organizational identification was stronger. However, the influences were non-existent when employee moral identity was high. Third, to further assess the presence of moderated mediation, we examined the index of moderated mediation obtained by PROCESS (Hayes, 2015). We found that moral identity moderated the indirect relationship between self-serving leadership and interpersonal deviance with an index of −0.02 (95% CI [−0.04, −0.00]), and it moderated the indirect relationship between self-serving leadership and organizational deviance with an index of −0.02 (95% CI [−0.04, −0.00]). Neither confidence interval included zero. Therefore, H4a and H4b were confirmed.

**TABLE 9:** Moderated mediation of interpersonal deviance and organizational deviance across levels of moral identity.

Level (Moderator)	SL – OI - WDB-I			SL – OI - WDB-O		
	Indirect effect	SE	95%CI	Indirect effect	SE	95%CI
Low MI	0.04	0.01	[0.02, 0.06]	0.04	0.01	[0.02, 0.06]
Middle MI	0.02	0.01	[0.01, 0.04]	0.02	0.01	[0.01, 0.04]
High MI	0.01	0.01	[−0.01, 0.03]	0.01	0.01	[−0.00, 0.03]

*N = 377. SL, self-serving leadership; OI, organizational identification; MI, moral identity; WDB-I, interpersonal deviance; WDB-O, organizational deviance. Low MI represents mean −1 SD, and high MI represents mean + 1 SD; S.E., standard error; BC, bias-corrected; CI, confidence interval.*

## Discussion

From social identity theory perspective, we propose a theoretical model that self-serving leadership induces employee interpersonal deviance and organizational deviance through organization identification, and we explore the moderating role of moral identity in this relationship. Based on survey data collected from 377 questionnaires by using a three-wave time lagged design, We get following conclusions:

First, The main effect of self-serving leadership on employees’ workplace deviance. Self-serving leadership positively correlated with employees’ interpersonal deviance and organizational deviance. In other words, the more strongly employees perceive the self-serving behaviors of leaders, the more deviance they will behave in the workplace. This is consistent with most research results on negative leadership behavior (Mitchell and Ambrose, 2007; Vogel and Mitchell, 2015; Zhou et al., 2021). According to social learning theory and negative reciprocal norms, employees will observe and imitate leaders’ self-serving behaviors by acquiring self-serving values. It can impact the organizational ethical climate in which members put their own interests first. When subordinates feel a sense of threat that their resources are exploited by the supervisor, they are inclined to take actions to restore the balance (Carlsmith et al., 2002). Thus, it induces deviant behavior of employees.

Second, we tested the mediating effect of organizational identification. The self-serving behavior of leaders in an organization will decrease employees’ identification with the organization, thus increasing employees’ deviance. According to Social identity theory, when the organization meets employees’ needs for security, self-realization, and belonging, individuals will classify themselves as members of that organization, and motivate employees’ positive attitudes and behavior to organization (Tajfel and Turner, 1986). Self-serving leadership, as an unethical leadership behavior (Peng et al., 2019), will make subordinates perceive the risk of exploitation (Liu et al., 2017) and control (Camps et al., 2012), It violates employee expectations of a safe work environment, respect, and fairness, which make them form a sense of exclusion by the organization and reduce their sense of belonging, and then harm their organizational identification, damage the emotions between employees and the organization, make employees indifferent to the organization (Ashforth and Mael, 1989), induce workplace deviance of employees.

Third, we tested moderating effect of moral identity. This research reveals that the negative effect between self-serving leadership and organizational identification employees is stronger for employees with low moral identity than for those with high moral identity. Previous studies have shown that high moral identity makes individuals constrained by their own moral codes (Mulder and Aquino, 2013). They will condemn behavior that violates their moral code (Bandura, 1991). Compared with employees with low moral identity, employees with high moral identity pay more attention to information related to morality (Reed et al., 2007). Individuals tend to categorize themselves into groups that match their values (De Roeck et al., 2014). When the moral information displayed by the organizational context is consistent with the moral values of employees, employees will be more identified with the organization (Wang et al., 2017). This study reveals that when employees with high moral identity perceive the moral information of the supervisor is inconsistent with their own moral code, they will not exacerbate their negative impact on organizational identification. This finding suggests that the inclusiveness and positive interpersonal orientation of employees with high moral identity can promote high-quality leader-member exchange, and enhance the understanding of leaders’ behavior, and thus incline to have a positive interpretation of leaders’ self-serving behavior. Meanwhile, culture plays an important role in the formation of individual moral cognition (Shao et al., 2011). Different from western culture, subordinates in Asian culture context may think that leaders’ selfish behaviors are not immoral, which reduces the sensitivity of employees with high moral identity to perceive leaders’ self-serving behaviors, thus alleviating the negative impact of self-serving leadership on employees’ organizational identification.

### Theoretical Implications

First, previous researches on the influence of self-serving leadership on employee behavior are mainly based on social exchange theory (e.g., Decoster et al., 2021) and social cognition theory (e.g., Ritzenhöfer et al., 2019). Few studies have explored the indirect relationship between self-serving leadership and employees’ workplace deviant behavior based on organizational identification. Organizational identification, as a sense of belonging to an organization, can effectively predict employees’ behaviors (Tajfel and Turner, 1986). Therefore, this study hypothesized that self-serving leaders have indirect negative effects on employees’ deviant behaviors through the mediating role of organizational identification, and conducted an empirical study to test it, which is a meaningful supplement to previous studies and enriches our understanding of the internal mechanism of self-serving leadership.

Second, this study proposes and tests the moderating role of moral identity as a boundary condition in the indirect relationship between organizational identification on self-serving leadership and deviant behavior. While Wang et al. (2017) found that when employees’ value is coherent with their organization, they will classify themselves into an organization and improve their organizational identification. Therefore, employees with high moral identity are more likely to classify themselves into an organization with ethical perception, and eventually feel a greater sense of organizational identification. According to this, when employees work with unethical leaders (e.g., self-serving leaders) who are not aligned with the values of employees, and amplifies negative perceptions of employees with high moral identity to the organizational identification. Our findings suggest that the effects may be more complicated. We consider the response of employee moral identity to leadership behavior in an Asian cultural contexts, and the moderating role of moral identity provides clues to our understanding how different cultures influence the effect of self-serving leadership. Although the research on self-serving leadership has gradually increased in recent years, most of the researches are still rooted in the western cultural background, and few studies on self-serving leadership considers different cultural backgrounds. This study, based on the influence of employee moral identity on the relationship between self-serving leadership and employee behavior in Asian cultural contexts, makes a theoretical contribution to the indigenous cultural study of self-serving leadership.

### Practical Implications

This paper discusses how self-serving leaders induce workplace deviance through the mediating role of organizational identification and the boundary conditions of moral identity. The research results may help organizations devise targeted measures to reduce workplace deviance and its negative impact.

First, organizations should take actions to prevent supervisors’ self-serving behavior. On the one hand, Some personality traits are related to self-serving behavior, such as narcissism (Nevicka et al., 2018). Therefore, to improve the selection mechanism of leaders, candidates with self-serving personality should be carefully examined. On the other hand, the organization should strengthen the supervision power of supervisors. Research shows that the more powerful is the leader, the more selfish will be his behavior (Bendahan et al., 2015). Therefore, organizations should guard against the negative effects of power, strengthen the system of effective restriction and supervision of power operation to prevent supervisors from abusing their power to seek improper interests. Finally, the specific department should take seriously to establish an interactive communication and feedback mechanism between employees and the organization to ensure that employees can feedback their opinions timely and effectively. Thus, employees can protect their legitimate rights and interests through formal channels rather than through deviant behaviors in the workplace.

Second, this research revealed the mediating role of organizational identification. Research shows that self-serving leadership can increase workplace deviance by undermining employees’ organizational identification. This reminds managers to pay attention to improving the quality of employment relationships to cultivate employees’ identification with the organization and a sense of belonging. On the one hand, the organization shall give full respect and trust to employees, affirm their value and contribution. On the other hand, it needs to care about employees’ lives and give them necessary help, which can make up for the weakening effect of self-serving leadership on employees’ organizational identification and reduce employees’ deviant behavior.

Finally, organizations should aim to enhance the level of employees’ moral identity. Moral identity is beneficial for organizations to cultivate employees’ organizational identification, enhance their sense of belonging to the organization, and improve their work enthusiasm (Van Dick et al., 2006). Therefore, we suggest that organizations pay closer attention to employees’ professional ethics and incorporate moral training into employees’ career development. Specifically, (1) moral identity should be used as a talent evaluation criterion, and candidates with high moral identity should be hired as often as possible; (2) organizations should train employees with weak moral identity to improve their moral identity; and (3) organizations should foster a positive, ethical corporate culture to promote the level of employees’ moral identity.

### Limitations and Future Research

Although this research has theoretical and practical significance, there are still some deficiencies. The first lies in data reporting. This study adopted the method of different times for data collection to reduce the effect of common method variance (CMV). However, due to the sensitive negative behavior surveyed in this study, there may be weaknesses or concealment with self-report data, as well as exaggeration of or hostility toward others’ evaluation in the report of such behavior, so the data may not resemble reality completely. Therefore, future research could consider the combination of leader-employee mutual evaluation and peer mutual evaluation, expand the data pool, and utilize multimethod assessment.

Second, there is a limitation regarding the findings on intermediary mechanisms. According to social identity theory, our research demonstrated that organizational identification take mediating role in the relationship between self-serving leadership and workplace deviance, but multiple variables may be inclined to influence the associate of self-serving leadership on employees’ workplace deviance (such as emotional factors). Therefore, future studies should explore the mediating mechanism from other perspectives.

Third, culture influences individuals’ beliefs, attitudes, behaviors, and values (Hofstede, 2001). In contrast with western cultures, Asians generally accept managers’ hostility to subordinates and maintain the hierarchy of supervisors and subordinates, so as to respect authority. Employees with a strong moral identity are less sensitive to the moral information contained in self-serving leadership, thereby reducing their intentions to engage in deviant behavior. This study examined the influence of self-serving leadership on employees’ attitudes and behaviors while considering cultural values. Future studies could extend this approach to explore specific cultural values, such as Confucianism, traditionalism, and collectivism.

Fourth, from the perspective of social identity, this study analyzes the influence of self-serving leadership on employees’ deviant behaviors. It only proves the influence of individual factors on employees’ deviant behaviors. However, work environment factors also play an important role in influencing employees’ behaviors (Salancik and Pfeffer, 1978). Therefore, future research can explore the influence of self-serving leadership on employees’ deviant behaviors from work environment factors, such as team ethical climate, team cultural norms, and team trust, to ensure systematic and comprehensive research.

## Conclusion

From the perspective of organizational identification, our research investigated the impact of self-serving leadership on employees’ workplace deviance. The present study adopted social identity theory to explore the impact of organizational identification on self-serving leadership and interpersonal deviance and organizational deviance. Through a longitudinal questionnaire survey of 377 employees, we found that self-serving leadership positively affects interpersonal deviance and organizational deviance. Furthermore, employees’ organizational identification make a mediating impact on this process. Additionally, employee with low moral identity can exacerbate the influence of self-serving leadership on interpersonal deviance and organizational deviance and further strengthen the mediating effect of organizational identification in the relationship of self-serving leadership on interpersonal deviance and organizational deviance. In conclusion, the empirical results reveal that self-serving leadership can induce employees’ deviant behavior. It is worth mentioning that individuals with strong moral identity can effectively resist the impact of negative leadership. The results help to enrich both trait and behavioral theories of leadership. With this research, we hope to lay the groundwork for future studies that are relevant to these topics.

## Data Availability Statement

The original contributions presented in the study are included in the article/supplementary material, further inquiries can be directed to the corresponding author.

## Author Contributions

LL and ZW: conceptualization and funding acquisition. YL and XW: data collection, writing, review, and editing. ZW: formal analysis and project administration. ZW and YL: writing – original draft preparation. All authors contributed to the article and approved the submitted version.

## Conflict of Interest

The authors declare that the research was conducted in the absence of any commercial or financial relationships that could be construed as a potential conflict of interest.

## Publisher’s Note

All claims expressed in this article are solely those of the authors and do not necessarily represent those of their affiliated organizations, or those of the publisher, the editors and the reviewers. Any product that may be evaluated in this article, or claim that may be made by its manufacturer, is not guaranteed or endorsed by the publisher.
